# Mapping Geological Events and Nitrogen Fixation Evolution Onto the Timetree of the Evolution of Nitrogen-Fixation Genes

**DOI:** 10.1093/molbev/msae023

**Published:** 2024-02-06

**Authors:** Hong-Wei Pi, Yin-Ru Chiang, Wen-Hsiung Li

**Affiliations:** Biodiversity Research Center, Academia Sinica, Taipei 115201, Taiwan; Department of Soil and Environmental Sciences, National Chung Hsing University, Taichung 40227, Taiwan; Biodiversity Research Center, Academia Sinica, Taipei 115201, Taiwan; Biodiversity Research Center, Academia Sinica, Taipei 115201, Taiwan; Department of Ecology and Evolution, University of Chicago, Chicago, IL 60637, USA

**Keywords:** biological nitrogen fixation, nitrogenase, timetree, geological event

## Abstract

Nitrogen is essential for all organisms, but biological nitrogen fixation (BNF) occurs only in a small fraction of prokaryotes. Previous studies divided nitrogenase-gene-carrying prokaryotes into Groups I to IV and provided evidence that BNF first evolved in bacteria. This study constructed a timetree of the evolution of nitrogen-fixation genes and estimated that archaea evolved BNF much later than bacteria and that nitrogen-fixing cyanobacteria evolved later than 1,900 MYA, considerably younger than the previous estimate of 2,200 MYA. Moreover, Groups III and II/I diverged ∼2,280 MYA, after the Kenorland supercontinent breakup (∼2,500–2,100 MYA) and the Great Oxidation Event (∼2,400–2,100 MYA); Groups III and Vnf/Anf diverged ∼2,086 MYA, after the Yarrabubba impact (∼2,229 MYA); and Groups II and I diverged ∼1,920 MYA, after the Vredefort impact (∼2,023 MYA). In summary, this study provided a timescale of BNF events and discussed the possible effects of geological events on BNF evolution.

## Introduction

Nitrogen is indispensable for all organisms, so nitrogen fixation is important for life on Earth. However, biological nitrogen fixation (BNF) occurs only in a small fraction of bacteria and archaea. BNF requires *nif* (nitrogen-fixing) genes, which include *nifH* (the dinitrogenase reductase subunit), *nifD* and *K* (the dinitrogenase subunits) and *nifE, N* and *B* (the FeMo-co biosynthetic subunits) (Rubio and Ludden 2005; Burén et al. 2020). *Nif* genes are usually present in a cluster in a genome (Dos Santos et al. 2012). The set of all six *nif* genes (*nifHDKENB*) was used to identify nitrogen-fixing species (Dos Santos et al. 2012) but some species with the five-gene set *nifHDKEB* or even the four-gene set *nifHDKB* have been found to be able to fix nitrogen (Zheng et al. 2016; Chen et al. 2021; Ivanovsky et al. 2021).

BNF was thought to have evolved first in archaea and later transferred to bacteria (the archaea-first hypothesis) (Raymond et al. 2004; Mus et al. 2019; Koirala and Brözel 2021), but we have recently provided strong evidence for the opposite view (i.e. the bacteria-first hypothesis) (Pi et al. 2022). However, there remain questions concerning the evolution of BNF such as when did nitrogen-fixing archaea and aerobic bacteria evolve, which have not been resolved by previous studies (Boyd et al. 2011; Koirala and Brözel 2021).

In this study, we first constructed the phylogeny of concatenated nitrogenase structural proteins, NifHDK, in species that carry at least four *nif* genes, and then used the RelTime method to construct a timetree to estimate the evolutionary dates of BNF in certain species groups, including archaea and aerobic bacteria. We also related geological events such as meteorites and continental breakups to the evolution of BNF because such events should have strong impacts on the evolution of many organisms.

## Results and Discussion

### Phylogeny of Concatenated NifHDK Proteins

**Fig. 1.**
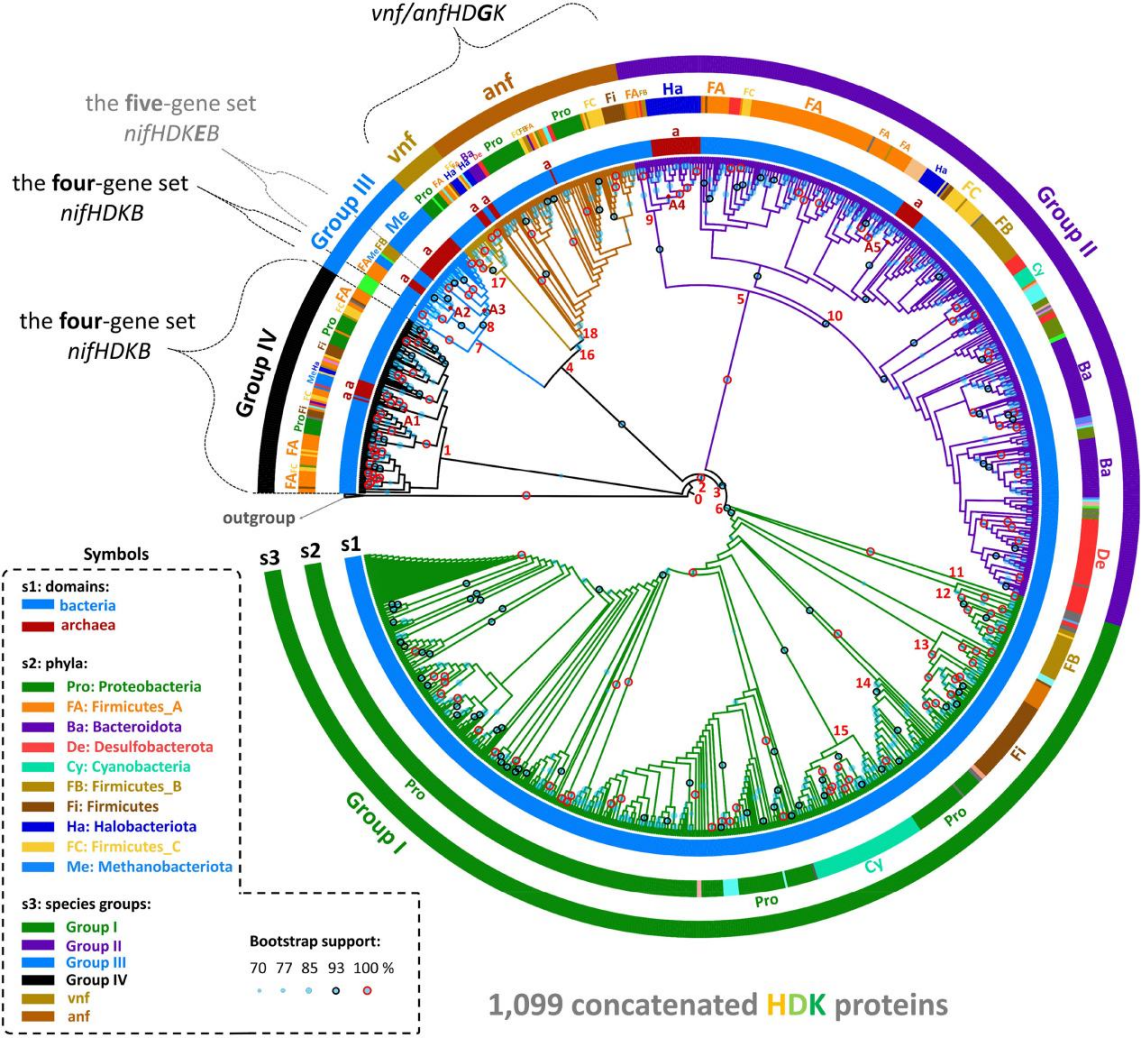
Phylogeny of 1,099 concatenated NifHDK protein sequences. The protein sequences were classified into Groups I, II, III, IV, Vnf, and Anf. The trees were constructed by the maximum likelihood method in MEGA X, and ModelFinder was used to select the best-fit model of protein sequence evolution (1,000 bootstrap replicates); the model selected was LG + G + I + F. The concatenated BchXYZ and Bch/Chl LNB sequences were used as the outgroup. The different sizes of blue solid circles denote the bootstrap values >70%, bootstrap values ≥93% were labeled by black outer rings and bootstrap values of 100% by red outer rings (see the symbol box). The bacterial sequences are labeled in blue and the archaeal sequences in dark red in the s1 circle. The top 10 largest phyla are labeled in different colors in the s2 circle. The different species groups of the phylogeny are labeled in different colors in the s3 circle. On the tree, the species with the four-gene set and those with the five-gene set are labeled, while those that are not marked have the six-gene set.

To study the evolution of BNF, we reconstructed the phylogenetic tree of 1,099 concatenated Nif/Vnf/AnfHDK protein sequences (Fig. 1); the sequence data are listed in supplementary Data S1, Supplementary Material online and supplementary fig. S1, Supplementary Material online is a detailed version of Fig. 1. We used 16 concatenated Bch/ChlLNB and BchXYZ proteins as the outgroup as in previous studies (Boyd and Peters 2013; Garcia et al. 2020). The tree consists of Groups I, II, III (with Vnf/Anf groups), and IV, with Group IV as the oldest group (Raymond et al. 2004; Pi et al. 2022). In Fig. 1, those tree branches with a bootstrap value >70% are indicated by solid circles of different sizes, and bootstrap values ≥93% are labeled by black outer rings and bootstrap values of 100% by red outer rings. The majority of our bootstrap values are >93% (see also supplementary fig. S1, Supplementary Material online). The topology of Fig. 1 is similar to those in previous studies (Boyd and Peters 2013; Garcia et al. 2020); (Pi et al. 2022). Since Fig. 1 uses more HDK sequences than previous studies and most of its nodes are supported by a high bootstrap value, it is suitable for constructing the timetree of *nif* genes.

In Fig. 1, the divergence and radiation nodes of each group are labeled according to the tree topology. The ancestral node of Groups I, II, III (with Vnf/Anf groups), and IV is node 0, the node of Groups I, II, and III is node 2 and so on up to node 16 (Fig. 1). The five groups of archaeal sequences (red diamonds A1–A5 in Fig. 1) are nested inside bacterial sequences in all Groups, indicating that they have evolved later than the bacterial sequences, supporting the bacteria-first hypothesis (Pi et al. 2022).

### Timetree of *nif* Gene Clusters

**Fig. 2.**
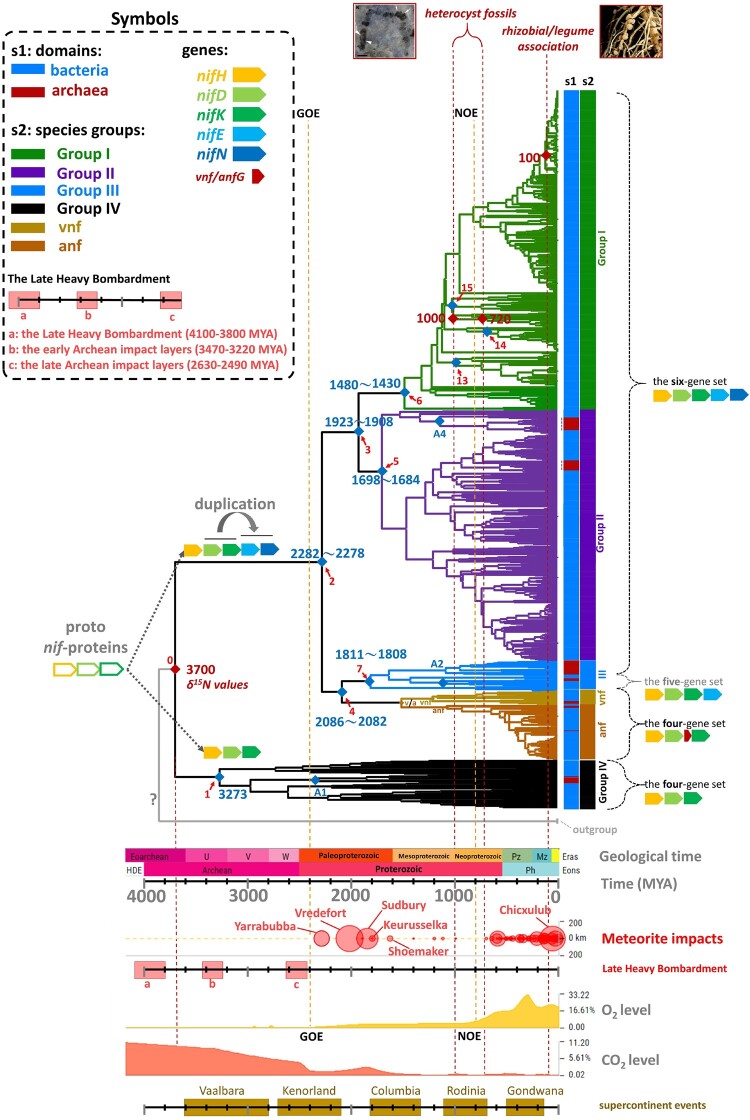
Timetree of nitrogenase gene clusters. The RelTime method in MEGA X was used to construct the timetree of concatenated NifHDK proteins. The BchXYZ and Bch/Chl LNB sequences were used as the outgroup. The estimated date for each branching node is indicated in MYA and the three reference dates (3,700, 1,000∼720, 100 MYA) were used to calibrate the timetree. The red diamonds represent the calibration points, while blue diamonds represent the estimated ages. This timetree is a combination of Timetrees 3 and 4 in Table 1. The geological age, meteorite impact date and size, O_2_ concentration, CO_2_ concentration, and supercontinental breakup date are given below the timetree. The bacterial sequences are labeled in blue, and the archaeal sequences in dark red in the s1 column. The different species groups of the phylogeny are labeled in different colors in the s2 column and branches.

We constructed ten timetrees from the phylogeny in Fig. 1 (Table 1); we provided 18 additional timetrees in supplementary Data S1 and S2, Supplementary Material online. We found that the branches of a subgroup of Group IV (the red branches in supplementary fig. S2, Supplementary Material online) were extremely short compared to the remaining branches, so we removed this subgroup from our analysis. We considered several calibration points, according to previous studies (Table 1 and supplementary Data S1, Supplementary Material online), and we described all our supplementary data, Supplementary Material online. The calibration points for heterocystous cyanobacteria (1,000–720 MYA) and rhizobia (100 MYA) are most reliable, because the former is based on fossil evidence (Pang et al. 2018), and the latter is based on the well-studied symbiotic relationship between modern legumes and rhizobia (Martin et al. 2017; van Velzen et al. 2019; Rutten et al. 2020). The calibration point for the origin of nitrogen fixation (3,700 MYA) is based on isotopic data of nitrogenase (Stüeken et al. 2021). We assumed that the common ancestor of all groups (node 0 in Fig. 1**)** was 3,700 MYA (Timetree 1), because all groups contain species that are known to be able to fix nitrogen (Zheng et al. 2016; Chen et al. 2021; Ivanovsky et al. 2021; Koirala and Brözel 2021). Figure 2 is based on these three calibration points (Timetrees 3 and 4 in Table 1). We also labeled the six-/five-/four-gene sets in Fig. 2.

**Table 1:** Evolutionary dates estimated from timetrees with different calibration time points

Date (MYA)	Timetree									
	1	2	3	4	5	6	7	8	9	10
Node 0-Groups I/II/III//IV	***3,700***	***4,280***	***3,700***	***3,700***	***4,280***	***3,950***	***3,800***	***3,470***	***3,700***	***3,700***
Node 1-Group IV	3,298	3,746	3,273	3,273	3,786	3,494	3,362	3,070	2,976	3,273
Node 2-Groups I/II/III	2,315	2,917	2,282	2,278	2,634	2,433	2,342	2,145	2,607	2,319
Node 3-Groups I/II	1,951	2,617	1,923	1,908	2,211	2,046	1,972	1,812	2,385	1,985
Node 4-Group III/Vnf/Anf	2,113	2,659	2,086	2,082	2,407	2,224	2,141	1,960	2,383	2,119
Node 5-Group II	1,723	2,195	1,698	1,684	1,952	1,806	1,741	1,600	2,105	1,752
Node 6-Group I	1,464	1,969	1,480	1,430	1,676	1,562	1,512	1,407	2,183	1,600
Node 7-Group III	1,834	2,434	1,811	1,808	2,090	1,931	1,859	1,702	2,069	1,841
Node 13	854	1,156	984	884	1,072	1,020	998	954	1,327	862
Node 14	522	592	672	575	716	690	679	658	1,293	819
Node 15-cyanobacteria	615	697	1,022	787	1,044	1,030	1,025	1,015	2,100	1,306
Heterocystous cyanobacteria	516	576	***1,000***	***720***	***1,000***	***1,000***	***1,000***	***1,000***	***2,100***	***1,300***
Rhizobia	57	44	***100***	***100***	***100***	***100***	***100***	***100***	***100***	***100***
A1 (IV)	2,407	2,850	2,332	2,332	2,698	2,490	2,395	2,187	2,121	2,332
A2 (III)	1,150	1,482	1,109	1,107	1,280	1,183	1,139	1,043	1,367	1,227
A4 (II)	1,158	1,445	1,136	1,127	1,306	1,208	1,164	1,070	1,232	1,096
Node 16-Vnf/Anf	1,509	1,781	1,508	1,505	1,741	1,608	1,548	1,417	1,723	1,532
Node 17-Vnf	1,206	1,492	1,209	1,207	1,396	1,289	1,241	1,136	1,382	1,229
Node 18-Anf	1,034	1,193	1,066	1,064	1,230	1,136	1,094	1,002	1,278	1,083

The time points indicated in Italics are the calibration points used for inferring the timetrees. For example, Timetree 4 was based on the calibration points: 3,700 MYA for node 0, 720 MYA for *Anhuithrix* (Pang et al. 2018), and 100 MYA for the emergence of rhizobia. In Timetrees 9 and 10, the calibration point for hetrocystous cyanobacteria were based on the isotopic dating (2,100–1,300 MYA) of the putative heterocystous cyanobacterium *Archaeoellipsoides* (Demoulin et al. 2019).

The date of node 0 would be older than 3,700 MYA if the common ancestor of Group IV (node 1) could not fix nitrogen. We have also constructed timetrees with several possible ancient dates of bacterial species from previous studies (Table 1): 4,280 MYA is the oldest putative fossil date of anaerobic Fe-oxidizing bacteria (Dodd et al. 2017; Lepot 2020; Papineau et al. 2022), 3,950–3,800 MYA are the upper and lower bounds of the isotope dating of Archean rocks (Arndt and Nisbet 2012; Tashiro et al. 2017), and 3,470 MYA is the dating of the oldest microbial mat-like structures in the Barberton greenstone belt (Hickman-Lewis et al. 2018). There seems to be a rate-slowdown in the evolution of Nif proteins as indicated by the observation that Timetree 1 gives a date of only 560 MYA for the node of heterocystous cyanobacteria and even Timetree 2 gives a date of 576 MYA, whereas the fossil date is ∼1,000–720 MYA (Pang et al. 2018). We constructed multiple timetrees, but some estimated time points are apparently too old or too young (supplementary Data S1 and S2, Supplementary Material online). The molecular clock test and the rate correlation test also reveal differences in BNF evolution rates (supplementary Data S1, Supplementary Material online). Thus, more recent events of BNF might have been underestimated.

We used the fossil record of 1,000–720 MYA for the existence of heterocystous cyanobacteria (*Anhuithrix*) (Pang et al. 2018) as the second calibration point and our estimate of the age of nitrogen-fixing cyanobacteria was ∼1,022–787 MYA (node 15, Fig. 2), which is much younger than the previous estimate (2,200 MYA) by analysis of antioxidant enzymes (Boden et al. 2021) and especially the dates (∼3,200–1,200 MYA) based on isotopic data of putative nitrogen-fixing cyanobacteria (Thomazo et al. 2018). Although there are some putative older heterocystous cyanobacteria fossils, such as ∼2,100–1,300 MYA for *Archaeoellipsoides* (Demoulin et al. 2019), they have been questioned as truly heterocystous cyanobacteria (Demoulin et al. 2019; Sánchez-Baracaldo et al. 2022). However, as our estimate is based on a single fossil record, which may not be the oldest, it may represent an underestimate. Based on these considerations, the actual age of nitrogen-fixing cyanobacteria may be somewhere between 1,100 and 2,200 MYA. Note that in Fig. 1, nitrogen-fixing cyanobacteria are nested within nitrogen-fixing proteobacteria and evolved later than nitrogen-fixing firmicutes in Group I, both of which should be younger than the common ancestor of Groups I/II (∼1,923 MYA). Thus, nitrogen-fixing cyanobacteria would be younger than 1,923 MYA.

Until 2022, the prevailing view was that BNF first evolved in archaea, but our timetree suggests that BNF evolved in archaea at most ∼2,332 MYA (node A1 in Fig. 2). This date is uncertain, because there is no experimental data to show that any archaeal species in Group IV is capable of nitrogen-fixation. Another possible date of BNF in archaea is node A2 (∼1,109 MYA), which implies that archaea-first gained BNF from thermophilic firmicutes in Group III (van Wolferen et al. 2013; Fuchsman et al. 2017). A third possible date is node A4 (∼1,136 MYA), which implies that archaea-first gained BNF from firmicutes to adapt to mesophilic environment (López-García et al. 2015). Both A2 and A4 possessed the set of all six *nif* genes and should be able to fix nitrogen. Both dates might have been underestimated because, as noted above, relatively recent events would tend to be underestimated.

### Relating Geological Events to Evolution of *nif* Genes

The evolution of BNF in cyanobacteria and other bacteria might have been significantly delayed by the Great Oxidation Event (GOE, ∼2,400–2,100 MYA; Lyons et al. 2014), because the function of nitrogenase is inhibited by oxygen. GOE would have strong negative effects on the evolution of BNF in most bacteria, although it might have no strong effect on prokaryotes living in anaerobic environments. The evolution of superoxide dismutase (SOD) and superoxide reductase (SOR) would have reduced the oxidative stress on a bacterium by converting the toxic superoxide into less toxic hydrogen peroxide. Previous studies suggested that these genes evolved because of GOE (Sheng et al. 2014; Case 2017). We indeed found that many nitrogen-fixing bacteria (in all groups) possess SOD/SOR genes (supplementary Data S1, Supplementary Material online, supplementary fig. S1, Supplementary Material online). Moreover, some cyanobacteria evolved special cells called “heterocysts”, which can provide an anaerobic environment for BNF (Sánchez-Baracaldo et al. 2022). On the other hand, the Neoproterozoic Oxidation Event (NOE, ∼800 MYA) (Lyons et al. 2014; Wallace et al. 2017) was suggested to have accelerated the evolution of life (including land plants and animals) on Earth (Knoll and Nowak 2017; Wallace et al. 2017; He et al. 2019). Interestingly, we found that some nitrogen-fixing aerobic bacteria (nodes 13–15 in Table 1) evolved near the beginning of NOE (Fig. 2). This may be because these bacteria already evolved the SOD genes, SOR genes, or heterocysts for mitigating oxidative stress, facilitating species radiation. The first nitrogen-fixing aerobic bacteria probably arose in aerobic firmicutes (node 13, ∼984–884 MYA), proteobacteria (node 14, ∼672–575 MYA), or cyanobacteria (node 15, ∼1,022–787 MYA) by gaining *nif* genes from anaerobic nitrogen-fixing bacteria. Note that each range of dates is estimated by the maximum and minimum date (1,000 and 720 MYA) of the *Anhuithrix* fossil (Pang et al. 2018).

In Fig. 2, the divergences of Groups I/II and III (∼2,282–2,278 MYA), Group III and Groups Vnf/Anf (∼2,086–2,082 MYA), and Groups I and II (∼1,923–1,908 MYA) occurred during or after the Kenorland supercontinent breakup (∼2,500–2,100 MYA) (Strand and Köykkä 2012). (A simplified version of Fig. 2 is given in supplementary fig. S3, Supplementary Material online.) It is possible that the breakup split bacterial populations into well separated smaller populations, facilitating speciation. Most species radiations in Groups I/II/III occurred after the Rodinia supercontinent breakup (∼1,130–750 MYA). In this connection, we note that it has been suggested that the evolution of ammonia-oxidizing archaea was related to the Rodinia supercontinent breakup (Yang et al. 2021).

There were several ancient meteorite impacts on Earth during the time period under consideration (Fig. 2), which might have created some isolated populations, facilitating speciation. Indeed, ancient meteorite impacts were proposed to have contributed to the evolution of ancient microbes (Lowe and Byerly 2018; Boden et al. 2021; Johnson et al. 2022). Note that the radiation of Group IV (∼3,273 MYA) occurred during the early Archean impact layers (Lowe and Byerly 2018) and the divergence between Groups III and I/II (node 2) occurred after the late Archean impact layers (Lowe and Byerly 2018). Interestingly, the common ancestor of Group IV species (node 1) is much older than the common ancestor of Groups I/II/III (node 2). The radiations in Groups I, II, and III (nodes 3–7) occurred after some well-defined meteorite impacts (Fig. 2) as described below. Recent studies suggested that the Yarrabubba impact (∼2,229 MYA) triggered large environmental changes (Erickson et al. 2020). The Vredefort (∼2,023 MYA), Sudbury (∼1,850 MYA), and Shoemaker (∼1,630 MYA) meteorite impacts might have strong impacts on the evolution of *nif* genes because they would have strongly changed earth's environments (Kring 2003; Pirajno et al. 2009; Erickson et al. 2020; Osinski et al. 2022). It has been suggested that multiple ancient meteorite impacts delayed the increase of atmospheric O_2_ (Marchi et al. 2021; Sánchez-Baracaldo et al. 2022) and even reduced the atmospheric O_2_ level to 1% after GOE (Sánchez-Baracaldo et al. 2022). This would have reduced the impacts of rising O_2_ level on the evolution of BNF.

Previous studies suggested that ancient bacteria were thermophilic (Weiss et al. 2016; Leng et al. 2023). We collected environmental data (supplementary Data S1, Supplementary Material online) of thermophilic bacteria from BacDive (Reimer et al. 2018) and NCBI BioSample (Barrett et al. 2012). Interestingly, there are many thermophilic bacteria in the early lineages of Groups I/II/III (Fig. 1). Moreover, the volcanic activities caused by the supercontinent breakup (Strand and Köykkä 2012), and the high temperature hydrothermal effluents and global thermal pools caused by ancient meteorite impacts (Lowe and Byerly 2018) might explain why the early lineages of our phylogeny are thermophilic bacteria, because they would have survived better under high temperatures than other bacteria. The evolution of *Roseiflexus* spp., which are thermophilic bacteria in the early lineages of Group III, was found to be related to ancient crustal changes (Gaisin et al. 2016).

## Methods

### Data Collection

Our data collection was as described previously (Pi et al. 2022); the data are given in supplementary Data S1, Supplementary Material online, and each dataset is described in Supplementary Material online. The nitrogenase proteins sequences and other genes were downloaded from the AnnoTree database (Mendler et al. 2019) based on the functional annotation of TIGRFAM (v15.0) and the homology information of KEGG (UniRef100). Each protein sequence has a gene ID (from AnnoTree database). We used the R95 version of GTDB, which contained not only 17,935 cultivated isolate genomes (56%) but also 1,870 isolate and environmental samples (6%), 11,661 metagenome-assembled genomes (MAGs, 37%) and 444 single-amplified genomes (SAGs, 1%). Therefore, about 38% to 44% of the genomes in the GTDB database R95 were from uncultured isolates (supplementary Data S1, Supplementary Material online). We also found that for the nitrogen-fixing species, our study included 43 cultivated archaeal genomes (68% in archaeal collection), 1 environmental archaeal sample (2%), and 19 archaeal MAGs (30%); and 692 cultivated bacterial genomes (82% in bacterial collection), 7 environmental bacterial samples (0.8%), 141 bacterial MAGs (17%), and 2 bacterial SAGs (0.2%).

### Phylogenetic Tree Reconstruction

The Nif protein sequences were aligned by MAFFT (v7.487) (Katoh and Standley 2013). The alignments of Nif protein sequences were trimmed using TrimAl (v1.3) (Capella-Gutiérrez et al. 2009; Sánchez et al. 2011) (using a heuristic selection of the automatic method based on similarity statistics). The alignments (after trimming) can be found in supplementary Data S3, Supplementary Material online. We used the MEGA X software (Kumar et al. 2018) to reconstruct maximum likelihood trees (Fig. 1), with 1,000 bootstrap replicates, in which the best-fit model of protein sequence evolution selected according to the Bayesian information criterion was used. Each phylogeny was annotated by the tool “Interactive tree of life v4” (Letunic and Bork 2019). Some additional descriptions of phylogenetic reconstruction are given in the figure legends.

### Timetree Construction

We used the maximum likelihood tree in Fig. 1 to construct the timetree by the RelTime method (Tamura et al. 2012, 2018) in MEGA X (Kumar et al. 2018). The branch lengths were calculated using the maximum likelihood method and the Le_Gascuel_2008 substitution model (Le and Gascuel 2008). A discrete Gamma distribution was used to model evolutionary rate differences among residue sites (five categories (+G, parameter = 0.7015)), allowing for some sites to be evolutionarily invariable ([ + I], 1.69% sites). The model was based on the best-fit model of Fig. 1. The timetrees were computed using calibration points listed in Table 1 and supplementary Data S1, Supplementary Material online. The raw timetree figures are given in supplementary Data S2, Supplementary Material online and have detailed information in supplementary Data S1, Supplementary Material online. The geological events are according to the TimeTree version 5 database (Kumar et al. 2022) and other references in discussion part.

## Supplementary Material

msae023_Supplementary_Data

## Data Availability

The data underlying this article are available in the article and in its online supplementary material.
