# Supplementary material for: Mapping Geological Events and Nitrogen Fixation Evolution Onto the Timetree of the Evolution of Nitrogen-Fixation Genes
Source: Mol Biol Evol. 2024 Feb 6;41(2):msae023. doi: 10.1093/molbev/msae023 (PMC10881105; doi:10.1093/molbev/msae023)
Supplement: msae023_Supplementary_Data [file msae023_supplementary_data.zip › Supplementary Fig. 3.pdf]

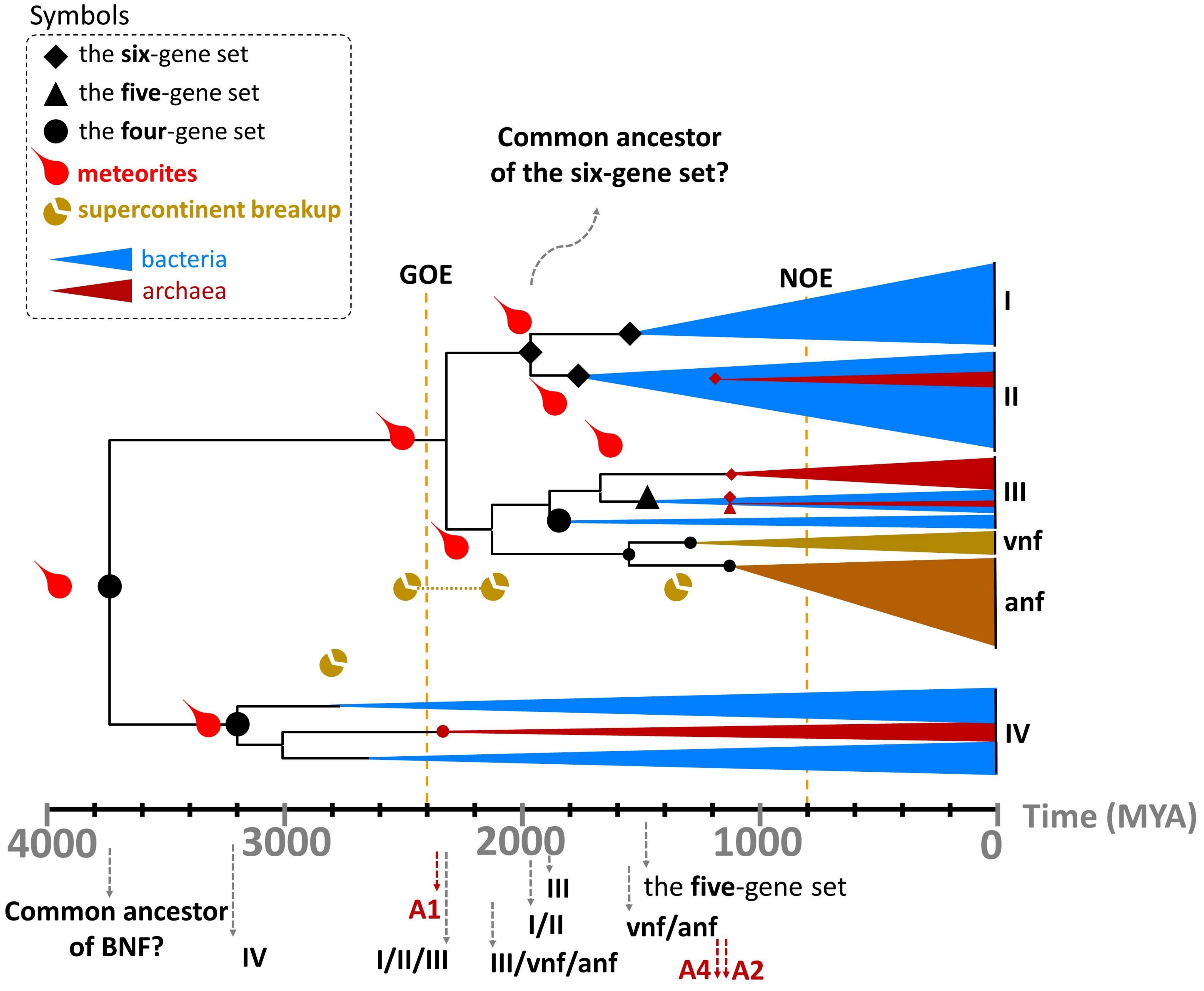
Symbols
the six-gene set
the five-gene set
the four-gene set
meteorites
supercontinent breakup
bacteria
archaea
Common ancestor
of the six-gene set?
GOE
NOE
I
II
III
vnf
anf
IV
4000
3000
2000
1000
0
Time (MYA)
Common ancestor
of BNF?
IV
A1
I/II/III
III/vnf/anf
I/II
III
the five-gene set
vnf/anf
A4
A2
