# Supplementary material for: Mapping Geological Events and Nitrogen Fixation Evolution Onto the Timetree of the Evolution of Nitrogen-Fixation Genes
Source: Mol Biol Evol. 2024 Feb 6;41(2):msae023. doi: 10.1093/molbev/msae023 (PMC10881105; doi:10.1093/molbev/msae023)
Supplement: msae023_Supplementary_Data [file msae023_supplementary_data.zip › Supplementary_information-20240129-final.pdf]

Mapping geological events and nitrogen fixation evolution onto the timetree of the evolution of nitrogen-fixation genes

Hong-Wei Pi[1,2,*] (hwpi1112@nchu.edu.tw)
Yin-Ru Chiang[1] (yinru915@gate.sinica.edu.tw)
Wen-Hsiung Li[1,3,*] (whli@uchicago.edu)

Affiliations

[1] Biodiversity Research Center, Academia Sinica, Taipei, Taiwan 115201

[2] Department of Soil and Environmental Sciences, National Chung Hsing University, Taichung 40227, Taiwan

[3] Department of Ecology and Evolution, University of Chicago, Chicago 60637, USA

[*] To whom correspondence should be addressed.

Corresponding author E-mail address:

whli@uchicago.edu (W-H Li), hwpi1112@nchu.edu.tw (H-W Pi)

This file includes:

Legends for Supplementary Figures, which are on separate PDF files:

Supplementary Fig. 1: The complete version of the phylogeny in Fig. 1 and the distribution of superoxide dismutase (SOD) and superoxide reductase (SOR) genes in the phylogeny of Fig. 1.

Supplementary Fig. 2: The comparison of the scaled tree of Fig. 1 and Timetree 1.

Supplementary Fig. 3: A simplified version of Fig. 2.

Supplementary Datasets:

Supplementary Data 1

Supplementary Data 2: The raw timetree figures analyzed using MEGA X.

Supplementary Data 3: The alignment file of Fig. 1.

Legends for Supplementary Figures (on separate PDF files):

Supplementary Fig. 1: The complete version of the phylogeny in Fig. 1

This figure provides the details of Fig. 1. It can be zoomed in for a clear view. The figure shows the bootstrap values (1=100%, 0.9=90%...). One can search the geneID, which is given in the fourth line of Supplementary Fig. 1. The geneID can be used to search for information in **Supplementary Data 1**. The identified SOD (SodA/B/C/N) and SOR (AhpC, Ccp, Dfx) genes in nitrogen-fixing bacteria in the phylogeny of Fig. 1. The detailed information is listed in **Supplementary Data 1**.

Supplementary Fig. 2: The comparison of the scaled tree in Fig. 1 and Timetree 1.

The RelTime method in MEGA X was used to construct the timetree of concatenated NifHDK proteins. The BchXYZ and Bch/Chl LNB sequences were used as the outgroup. This is the original topology of Fig. 1 and Timetree 1. The subgroup of branches (in red color) with very short branch lengths is deleted before running the RelTime analysis; that is, the deleted subgroup is marked in red color. The clade removed only consisted of bacteria, most of which are Firmicutes_A. It is in the first subgroup of Group IV in **Supplementary Fig. 1.**

Supplementary Fig. 3: A simplified version of Fig. 2

We label the six-gene set by a diamond, the five-gene set by a triangle symbol, and the four-gene set by a circle. The meteorite impacts and supercontinent breakups are also labeled in fig. 2 at their geological date and related BNF events.

Supplementary Datasets:

Supplementary Data 1 (in Excel files): Below we explain the data meaning of each page in this file.

1. GTDB taxonomy: The taxonomic information of the 842 bacterial species and 63 archaeal species included in this study. We collect the bacterial and archaeal carrying the six-gene set, the five-gene set or the four-gene set.

2. Species inf1: All data information for Figs. 1 and 2. One can search the "GTDB genome ID" in GTDB (https://gtdb.ecogenomic.org/) for detailed information of the genome. The "ncbi_taxid" is for comparing the species name between GTDB and NCBI. We labeled the six-gene set, the five-gene set or the four-gene set in the "HDKENB column". The "group of nitrognease" is a comparison of our topology and those of

previous studies. We search for HDK sequences based on whether there is a closely linked NifDK behind the NifH in a genome, so we search the NifH sequence IDs "nifH gene ID" from the AnnoTree database.

3. Species inf2: The thermophilic bacteria in the early lineages of Groups III/II/I.
To identify the thermophilic species in our collection, we collect the data from BacDive and NCBI BioSample. One can search the "ncbi_biosample" in NCBI (https://www.ncbi.nlm.nih.gov/biosample).

4. R95 genome category: Each genome category of GTDB R95.

5. Nif gene IDs: The Nif protein sequence IDs of each species
As described above, we collect the closely linked HDK sequences, and we list them in this page.

6. All Nif sequences: The Nif protein sequences. This is the raw data that we downloaded from AnnoTree database.

7. Outgroup of the phylogeny: The outgroup sequences of phylogenetic trees in this study. The outgroup genes, Bch/ChlLNB and BchXYZ, are well-studied[1-3] and were downloaded from AnnoTree database.

8. Supplementary Data 2: The references and estimated ages of each timetree under study and the results of the Maximum Likelihood molecular clocks test and the rate correlation test. Below, we explain each timetree in this page.

(1). TimeTree 1: This timetree is based on one calibration point 3700 MYA (the isotopic data for BNF)[4] for node 0, the common ancestor of Groups I/II/III/IV.

(2). TimeTree 2: This timetree is based on the calibration point of 4280 MYA (the oldest putative fossil date of anaerobic Fe-oxidizing bacteria)[5] for node 0, the common ancestor of Groups I/II/III/IV.

(3). TimeTree 3: This timetree is based on three calibration points: 3700 MYA[4] for node 0, the common ancestor of Groups I/II/III/IV; 1000 MYA[6] for heterocystous cyanobacteria *Anhuithrix*; and 100 MYA[7] for the emergence of rhizobia.

(4). TimeTree 4: This timetree is based on three calibration points: 3700 MYA[4] for node 0, the common ancestor of Groups I/II/III/IV; 720 MYA[6] for heterocystous cyanobacteria *Anhuithrix*; and 100 MYA[7] for the emergence of rhizobia.

(5). TimeTree 5: This timetree is based on three calibration points: 4280 MYA (the

oldest putative fossil date of anaerobic Fe-oxidizing bacteria)[5] for node 0, the common ancestor of Groups I/II/III/IV; 1000 MYA[6] for heterocystous cyanobacteria *Anhuithrix*; and 100 MYA[7] for the emergence of rhizobia.

(6). TimeTree 6: This timetree is based on three calibration points: 3950 MYA (the isotope dating of Archean rocks)[8] for node 0, the common ancestor of Groups I/II/III/IV; 1000 MYA[6] for heterocystous cyanobacteria *Anhuithrix*; and 100 MYA[7] for the emergence of rhizobia.

(7). TimeTree 7: This timetree is based on three calibration points: 3800 MYA (the isotope dating of Archean rocks)[9,10] for node 0, the common ancestor of Groups I/II/III/IV; 1000 MYA[6] for heterocystous cyanobacteria *Anhuithrix*; and 100 MYA[7] for the emergence of rhizobia.

(8). TimeTree 8: This timetree is based on three calibration points: 3470 MYA (the dating of the oldest microbial mat-like structures in the Barberton greenstone belt)[11] for node 0, the common ancestor of Groups I/II/III/IV; 1000 MYA[6] for heterocystous cyanobacteria *Anhuithrix*; and 100 MYA[7] for the emergence of rhizobia.

(9). TimeTree 9: This timetree is based on three calibration points: 3700 MYA[4] for node 0, the common ancestor of Groups I/II/III/IV; 2100 MYA[12] for heterocystous cyanobacteria *Archaeoellipsoides*; and 100 MYA[7] for the emergence of rhizobia.

(10). TimeTree 10: This timetree is based on three calibration points: 3700 MYA[4] for node 0, the common ancestor of Groups I/II/III/IV; 1300 MYA[12] for heterocystous cyanobacteria *Archaeoellipsoides*; and 100 MYA[7] for the emergence of rhizobia.

(11). SD-TimeTree 1: This timetree (without removing a subgroup of Group IV in Supplementary Fig. 2) is based on the calibration point of 3700 MYA (the isotopic data for BNF)[4] for node 0, the common ancestor of Groups I/II/III/IV.

(12). SD-TimeTree 2: This timetree is based on the calibration point of 4280 MYA (the oldest putative fossil date of anaerobic Fe-oxidizing bacteria)[5] for node 0, the common ancestor of Groups I/II/III/IV.

(13). SD-TimeTree 3: This timetree is based on the calibration point of 1000 MYA[6] for heterocystous cyanobacteria *Anhuithrix*.

(14). SD-TimeTree 4: This timetree is based on the calibration point of 720 MYA[6] for heterocystous cyanobacteria *Anhuithrix*.

(15). SD-TimeTree 5: This timetree is based on the calibration point of 100 MYA[7] for the emergence of rhizobia.

(16). SD-TimeTree 6: This timetree is based on the calibration point of 3200 MYA (the isotopic data for molybdenum-nitrogenase)[13] for node 2, the common

ancestor of Groups I/II/III.

(17).SD-TimeTree 7: This timetree is based on the calibration point of 2200 MYA (the archaea-first hypothesis)[3] for A1, the archaeal Group IV NifHDK.

(18).SD-TimeTree 8: This timetree is based on the calibration point of 2200 MYA (the common ancestor of nitrogen-fixing cyanobacteria)[14], which was suggested by the cyanobacterial antioxidant enzymes.

(19).SD-TimeTree 9: This timetree is based on two calibration points: 3700 MYA[4] for node 0, the common ancestor of Groups I/II/III/IV; and 100 MYA[7] for the emergence of rhizobia.

(20).SD-TimeTree 10: This timetree is based on two calibration points: 3700 MYA[4] for node 0, the common ancestor of Groups I/II/III/IV; and 2200 MYA (the common ancestor of nitrogen-fixing cyanobacteria)[14], which was suggested by the cyanobacterial antioxidant enzymes.

(21).SD-TimeTree 11: This timetree is based on two calibration points: 2200 MYA (the common ancestor of nitrogen-fixing cyanobacteria)[14], which was suggested by the cyanobacterial antioxidant enzymes; and 100 MYA[7] for the emergence of rhizobia.

(22).SD-TimeTree 12: This timetree is based on two calibration points: 3700 MYA[4] for node 0, the common ancestor of Groups I/II/III/IV; and 2200 MYA (the archaea-first hypothesis)[3] for A1, the archaeal Group IV NifHDK.

(23).SD-TimeTree 13: This timetree is based on two calibration points: 3700 MYA[4] for node 0, the common ancestor of Groups I/II/III/IV; and 2200 MYA (the archaea-first hypothesis)[3] for A2, the archaeal Group III NifHDK.

(24).SD-TimeTree 14: This timetree (without removing a subgroup of Group IV in Supplementary Fig. 2) is based on three calibration points: 3700 MYA[4] for node 0, the common ancestor of Groups I/II/III/IV; 1000 MYA[6] for heterocystous cyanobacteria *Anhuithrix*; and 100 MYA[7] for the emergence of rhizobia.

(25).SD-TimeTree 15: This timetree (without removing a subgroup of Group IV in Supplementary Fig. 2) is based on three calibration points: 3700 MYA[4] for node 0, the common ancestor of Groups I/II/III/IV; 720 MYA[6] for heterocystous cyanobacteria *Anhuithrix*; and 100 MYA[7] for the emergence of rhizobia.

(26).SD-TimeTree 16: This timetree is based on three calibration points: 3700 MYA[4] for node 0, the common ancestor of Groups I/II/III/IV; 1480 MYA[12] for heterocystous cyanobacteria *Archaeoellipsoides*; and 100 MYA[7] for the emergence of rhizobia.

(27).SD-TimeTree 17: This timetree is based on three calibration points: 3700 MYA[4] for node 0, the common ancestor of Groups I/II/III/IV; 3200 MYA(the isotopic data for molybdenum-nitrogenase)[13] for node 2, the common ancestor of

Groups I/II/III; and 100 MYA[7] for the emergence of rhizobia.

(28). SD-TimeTree 18: This timetree is based on three calibration points: 3700 MYA[4] for node 0, the common ancestor of Groups I/II/III/IV; 720 MYA[6] for heterocystous cyanobacteria *Anhuithrix*; and 100 MYA[7] for the emergence of rhizobia.

The molecular clock test and the rate correlation test (analyzed by MEGA X [15]) suggested different evolutionary rates of Nif proteins.

9. SOD and SOR: The superoxide dismutase enzymes (SodA/B/C/N) and oxidative stress genes (AhpC, Ccp, Dfx) in our nitrogen-fixing bacteria.

Supplementary Data 2: The raw timetree figures analyzed using MEGA X. These timetree analysis results are described in Supplementary Data 1.

Supplementary Data 3: The alignment file of Fig. 1.

References:

1 Garcia, A. K., McShea, H., Kolaczkowski, B. & Kaçar, B. Reconstructing the evolutionary history of nitrogenases: Evidence for ancestral molybdenum-cofactor utilization. *Geobiology* **18**, 394-411 (2020).

2 Boyd, E. & Peters, J. W. New insights into the evolutionary history of biological nitrogen fixation. *Front Microbiol* **4**, 201 (2013).

3 Boyd, E. *et al.* A late methanogen origin for molybdenum-dependent nitrogenase. *Geobiology* **9**, 221-232 (2011).

4 Stüeken, E. E., Boocock, T., Szilas, K., Mikhail, S. & Gardiner, N. J. Reconstructing Nitrogen Sources to Earth's Earliest Biosphere at 3.7 Ga. *Frontiers in Earth Science* **9**, 286 (2021).

5 Papineau, D. *et al.* Metabolically diverse primordial microbial communities in Earth's oldest seafloor-hydrothermal jasper. *Science Advances* **8**, eabm2296 (2022).

6 Pang, K. *et al.* Nitrogen-fixing heterocystous cyanobacteria in the Tonian period. *Current Biology* **28**, 616-622. e611 (2018).

7 Martin, F. M., Uroz, S. & Barker, D. G. Ancestral alliances: plant mutualistic symbioses with fungi and bacteria. *Science* **356**, eaad4501 (2017).

8 Tashiro, T. *et al.* Early trace of life from 3.95 Ga sedimentary rocks in Labrador, Canada. *Nature* **549**, 516-518 (2017).

9 Arndt, N. T. & Nisbet, E. G. Processes on the young Earth and the habitats of early life. *Annual Review of Earth and Planetary Sciences* **40**, 521-549 (2012).

10 Weiss, M. C. *et al.* The physiology and habitat of the last universal common ancestor. *Nature microbiology* **1**, 1-8 (2016).

11 Hickman-Lewis, K., Cavalazzi, B., Foucher, F. & Westall, F. Most ancient evidence for life in the Barberton greenstone belt: Microbial mats and biofabrics of the~ 3.47 Ga Middle Marker horizon. *Precambrian Research* **312**, 45-67 (2018).

12 Demoulin, C. F. *et al.* Cyanobacteria evolution: Insight from the fossil record. *Free Radical Biology and Medicine* **140**, 206-223 (2019).

13 Stüeken, E. E., Buick, R., Guy, B. M. & Koehler, M. C. Isotopic evidence for biological nitrogen fixation by molybdenum-nitrogenase from 3.2 Gyr. *Nature* **520**, 666 (2015).

14 Boden, J. S., Konhauser, K. O., Robbins, L. J. & Sánchez-Baracaldo, P. Timing the evolution of antioxidant enzymes in cyanobacteria. *Nature Communications* **12**, 4742 (2021).

15 Kumar, S., Stecher, G., Li, M., Knyaz, C. & Tamura, K. MEGA X: molecular evolutionary genetics analysis across computing platforms. *Molecular biology and evolution* **35**, 1547 (2018).
