# Supplementary figures and images for: Mapping Geological Events and Nitrogen Fixation Evolution Onto the Timetree of the Evolution of Nitrogen-Fixation Genes
Source: Mol Biol Evol. 2024 Feb 6;41(2):msae023. doi: 10.1093/molbev/msae023 (PMC10881105; doi:10.1093/molbev/msae023)

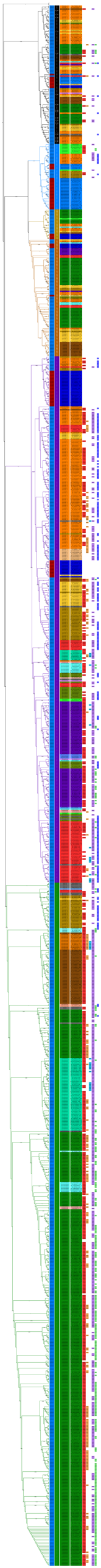

Supplement: msae023_Supplementary_Data [file msae023_supplementary_data.zip › Supplementary Fig. 1.pdf]

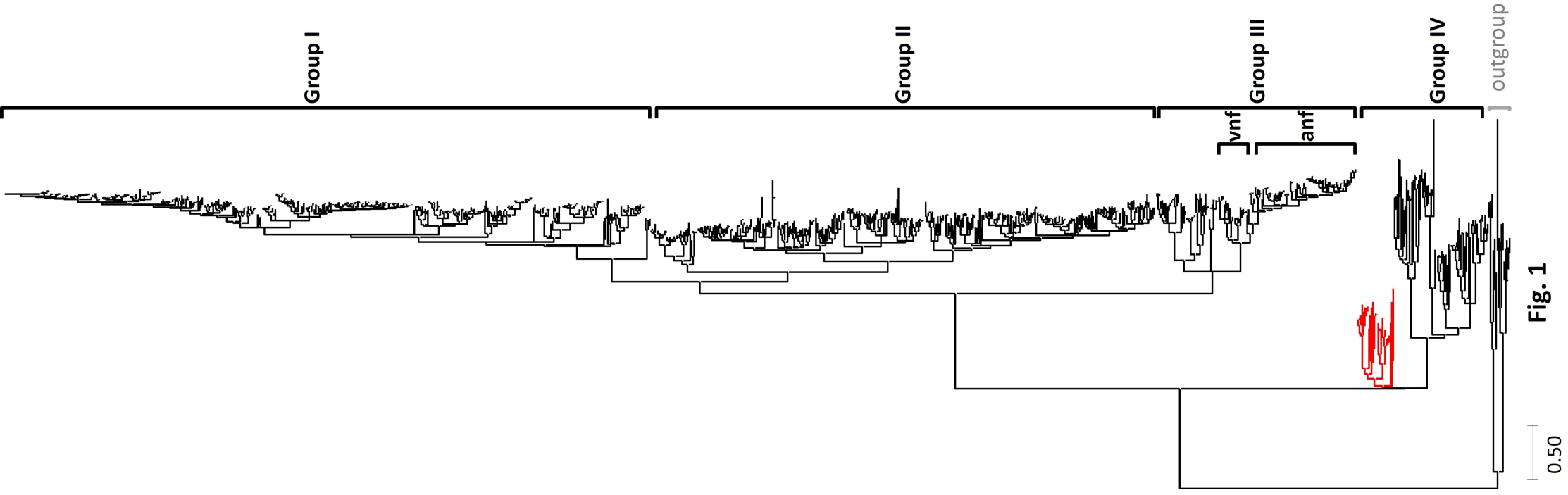

Fig. 1

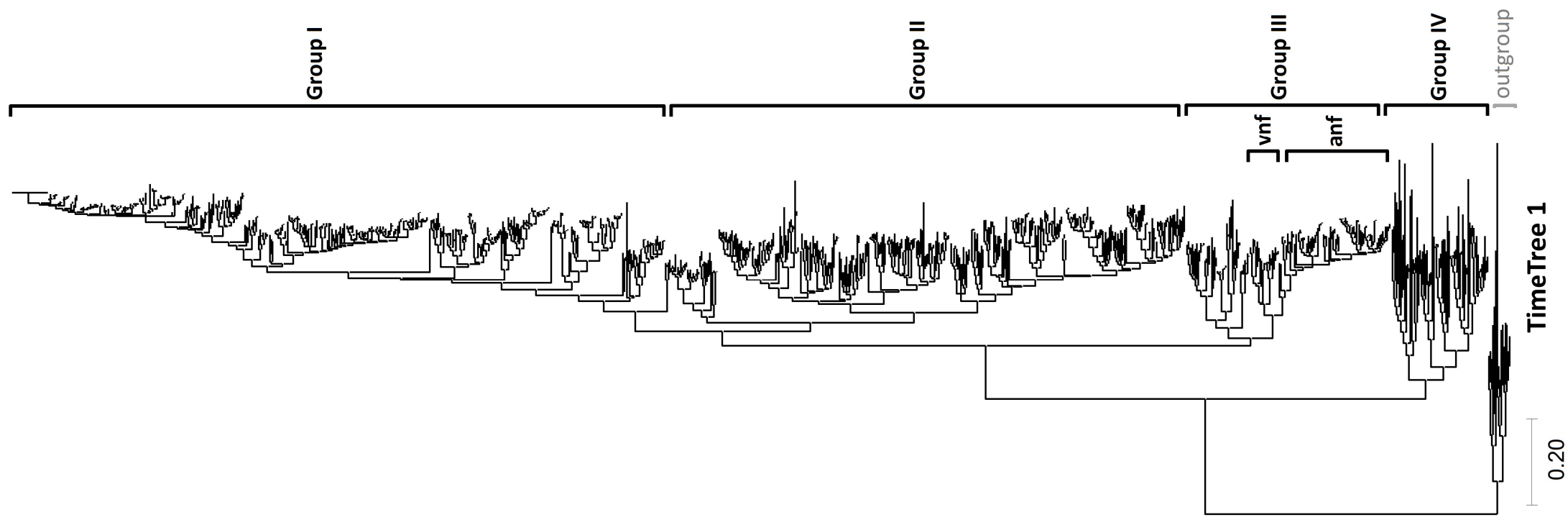

TimeTree 1

Supplement: msae023_Supplementary_Data [file msae023_supplementary_data.zip › Supplementary Fig. 2.pdf]
